# Pharmacokinetics of plasma lopinavir and ritonavir in tuberculosis–HIV co-infected African adult patients also receiving rifabutin 150 or 300 mg three times per week

**DOI:** 10.1186/s12941-020-0345-6

**Published:** 2020-01-22

**Authors:** Henri Gautier Ouedraogo, Alberto Matteelli, Giorgia Sulis, Tegwinde Rebeca Compaore, Serge Diagbouga, Simon Tiendrebeogo, Alberto Roggi, Kadari Cisse, Pier Francesco Giorgetti, Paola Villani, Lassana Sangare, Jacques Simpore, Mario Regazzi, Seni Kouanda

**Affiliations:** 1 Biomedical Research Laboratory, Institut de Recherche en Sciences de la Santé (IRSS), 03BP7192, Ouagadougou, Burkina Faso; 2 Institute of Infectious and Tropical Diseases, Brescia University Hospital, Brescia, Italy; 3 Department of Epidemiology, Biostatistics and Occupational Health, McGill University, Montreal, QC Canada; 4 McGill International TB Centre, McGill University, Montreal, QC Canada; 5 Laboratory of Clinical Pharmacokinetics, IRCCS - San Matteo University Hospital, Pavia, Italy; 6 Laboratory of Virology, CHU-Yalgado Ouedraogo, Ouagadougou, Burkina Faso; 7 Centre de Recherche Biomoléculaire Pietro Annigoni (CERBA), Ouagadougou, Burkina Faso

**Keywords:** Pharmacokinetic, Lopinavir, Ritonavir, Rifabutin, HIV–tuberculosis, Co-infection

## Abstract

**Background:**

To evaluate the pharmacokinetic of plasma lopinavir (LPV) and ritonavir (RTV) when co-administered with three times weekly (TPW) rifabutin (RBT) at a dose of either 150 or 300 mg in African tuberculosis (TB) and HIV co-infected adult patients.

**Methods:**

This is a pharmacokinetic study conducted in Ouagadougou among patients treated with a standard dosage of LPV/RTV 400/100 mg twice daily and RBT 150 mg TPW (arm A = 9 patients) or rifabutin 300 mg TPW (arm B = 7 patients) based regimens. Patients were recruited from the Bogodogo and Kossodo district hospitals in Ouagadougou from May 2013 to December 2015. Study inclusion criteria were that the patients were between 18 and 60 years of age, HIV-1 infected with pulmonary tuberculosis confirmed or suspected. Subsequent blood samples for pharmacokinetic monitoring were collected at 1, 2, 3, 4, 6, 8 and 12 h after combined drug ingestion for plasma drug monitoring using HPLC/MS assays.

**Results:**

The medians LPV C_max_ and T_max_ were respectively, 20 μg/mL and 4 h for the RBT 150 mg group (arm A) and 7.7 μg/mL and 3 h for the RBT 300 mg group (arm B). The AUC_0–12_ of LPV was 111.8 μg h/mL in patients belonging to arm A versus 69.9 μg/mL for those in arm B (p = 0.313). The C_0_ of LPV was lower than 4 μg/mL in three patients receiving RBT 300 mg. Of note, the RTV plasma concentrations were nearly halved among patients on RBT 300 mg compared to those on lower RBT doses. The AUC_0–12_ of RTV in arm A was 12.7 μg h/mL versus 6.6 μg h/ml in arm B (p = 0.313).

**Conclusion:**

In our study, the pharmacokinetic of LPV and RTV was found to be highly variable when coadministrated with RBT 150 mg or 300 mg three times per week. There is a need for specific large study to verify clinical and virological effects of this variation, especially when coadministrated with RBT of 300 mg TPW, and to prevent viral resistance in response to under-dosing of LPV.

*Trial registration* PACTR201310000629390. Registered 28 October 2013, http://www.pactr.org/

## Background

HIV/AIDS and tuberculosis (TB) both remain global public health problems, causing illness and the death of millions of people each year [1, 2]. TB is the most important AIDS-related opportunistic disease and is the leading cause of HIV/AIDS-related mortality in Sub-Saharan Africa. The risk of developing TB is estimated to be between 26 and 31 times greater in people living with HIV (PLHIV) than that of uninfected individuals [1].

Rifamycins are the core drugs of standard TB treatment regimens, irrespective of the patient’s HIV status. The clinical management of TB in HIV-infected patients receiving antiretroviral therapy (ART) can be complex for several reasons. Important drug interactions between rifamycins and protease inhibitors (PIs), such as lopinavir/ritonavir (LPV/RTV), which is still widely used in most resource-constrained settings, represent one of the most critical issues for clinicians [3, 4]. In fact, rifamycins are potent inducers of the cytochrome P450 pathway, in particular the CYP3A4 isoform, which is involved in the hepatic metabolism of many drugs including PIs thus leading to a reduction of their plasma concentrations, which may cause HIV treatment failure and favour the development of drug resistance [5–9]. On the other hand, PIs are also inhibitors of CYP3A and thus determine the accumulation of rifamycins, causing an increased risk of toxicity [10].

Rifabutin (RBT), is a derivative of rifamycins with a less potent inducer of CYP3A4 [11, 12]. It is recommended at 300 mg daily as prophylaxis and treatment of *Mycobacterium avium complex* (*MAC*) and for the treatment of drug susceptible tuberculosis [13]. Plasma concentrations of RBT are increased in the presence of protease inhibitors therefore dose adjustments are recommended when it is combined with a PI [11, 13–15].

Several dosages of RBT have been proposed to be used in combination with the standard dosage of LPV/RTV 400/100 mg twice daily, including thrice weekly RBT 150 mg [12], thrice weekly RBT 300 mg or once daily RBT 150 mg [3, 16, 17]. Some studies have assessed the pharmacokinetic profile of different doses of RBT under these conditions [18–21].

Achieving adequate plasma concentrations of LPV is essential to ensure a virological response and to prevent the selection of resistant viral mutants [22, 23].

The current recommendation is that RBT can be given with LPV/RTV without dose adjustment. However, data on the plasma concentration of ritonavir (RTV)-boosted LPV when co-administered with different doses of RBT are scarce. In a study evaluating the pharmacokinetic of RBT 150 mg thrice weekly or RBT 150 mg daily in African HIV—infected tuberculosis patients on LPV/RTV—based antiretroviral therapy, the authors reported that the median LPV trough (C_0_) concentrations were above the recommended lower limit for ART-naïve patients of 1 μg/mL [24]. Although there was a trend to higher LPV concentrations with the once daily dosing of RBT, the differences in AUC_0–12_ and C_max_ between the two doses were not significant [13]. In another study in Italy, the LPV serum concentrations were not reduced when the drug is administered together with an adjusted dose of RBT 150 mg thrice weekly [25].

Experience with RBT use for routine tuberculosis treatment is very limited in resource limited settings, particularly in Africa [13, 26], but the increasing number of patients on PI-based ART highlights the crucial role of this molecule in the therapeutic management of co-infected patients because rifampicin and LPV/RTV cannot be coadministered. Our study aimed to evaluate the plasma pharmacokinetics of LPV/RTV (400/100 mg) co-administered with RBT at a dosage of either 150 or 300 mg thrice weekly in TB/HIV co-infected adult patients in Burkina Faso.

## Methods

### Study design

This was a pharmacokinetic study based on the use of LPV and RTV in HIV and tuberculosis co-infected adults. They were being treated with rifabutin 150 mg thrice weekly or rifabutin 300 mg thrice weekly.

### Patients and study treatment

The patients were participating in the RIFLOPI study registered on PACTR201310000629390. They were recruited from the Bogodogo and Kossodo district hospitals in Ouagadougou from May 2013 to December 2015. Study inclusion criteria were that the patients were between 18 and 60 years of age, HIV-1 infected with pulmonary tuberculosis confirmed or suspected. That the patients were undergoing combined antiretroviral and tuberculosis treatment including a LPV/RTV standard regimen, as well as rifabutin 150 mg thrice weekly or rifabutin 300 mg thrice weekly for at least 2 weeks, and had given informed consent. The 2 weeks minimum delay comes from the time frame of building up full induction effect. The participating patients were divided into two groups. The first group (RBT 150 mg thrice weekly) consisted of nine patients on antiretroviral and anti-tuberculosis treatment including LPV (LPV/RTV 400/100 mg + 2INTI) twice daily in combination with rifabutin 150 mg thrice weekly and standard ethambutol–isoniazid–pyrazinamide. The second group (RBT 300 mg thrice weekly) consisted of seven patients treated with (LPV/RTV 400/100 mg + 2INTI) twice daily in combination with rifabutin 300 mg thrice weekly and standard ethambutol–isoniazid–pyrazinamide. Tuberculosis and HIV treatments were administered using the directly observed treatment, short-course (DOTS) strategy, and the national guidelines were used for HIV monitoring. Each patient took a daily dose of cotrimoxazole to prevent opportunistic infections associated with HIV.

### Pharmacokinetic monitoring

Pharmacokinetic monitoring was performed after 2 weeks of combined LPV/RTV and RBT treatment. On the day before pharmacokinetic monitoring, patients were admitted and fasted from midnight onwards. The pharmacokinetic assessment was conducted on a day when a dose of RBT was taken. The first measure of the pharmacokinetic monitoring (time zero) was performed on an empty stomach before taking the daily dose of RBT and LPV/RTV. After the first blood sampling, patients immediately (within 5 min) took their rifabutin and LPV/RTV regimen. Subsequent blood samples for pharmacokinetic monitoring were collected at 1, 2, 3, 4, 6, 8 and 12 h after combined drug ingestion. Breakfast (a sandwich and water) was served to the patients 2 h after drug ingestion.

Two to three millilitres of blood was collected in a heparinized primary vial and centrifuged at 3000 rpm for 10 min within 1 h of collection. The plasma was stored at − 20 °C until transportation to the laboratory for the pharmacokinetic analysis.

A high-performance liquid chromatography–mass spectrometry (HPLC/MS–MS) assay previously described by Moyer et al. [27] was used to determine the LPV and RTV plasma concentrations at the Service of Clinical Pharmacology (IRCCS S Matteo, Pavia, Italy). The limit of quantification was 0.05 µg/mL for both drugs. The assay was validated in accordance with the European Medicines Agency (EMA) “Guidelines on bioanalytical method validation [28]. The areas under the plasma concentration–time curve (AUC) were calculated by using the linear trapezoidal rule.

### Data management and analysis

Data were entered using EpiData (http://www.epidata.dk) and Excel and analysed with Stata, version 13 (https://www.stata.com, StataCorp LP; College Station, TX, USA). Descriptive statistics were used to describe the patient’s characteristics and to calculate the frequencies, proportions and medians with interquartile intervals. Statistical comparisons were made using Fisher’s exact test with 5% set as the significance level. For the LPV and RTV pharmacokinetic profile, we determined for each patient the C_max_ (peak concentration measured (µg/mL)), the C_trough_ (drug plasma concentration measured just prior to administration of the drug dose (µg/mL)), the T_max_ (time for C_max_), the area under the curve during a dosing interval (AUC_0–12_ = plasma concentration * time (µg × h/mL) and the apparent clearance (CL/F, true clearance divided by the true absolute bioavailability).

### Ethics issues

The study protocol was approved by the National Ethics Committee for Health Research and the national regulatory authority in Burkina Faso. All patients provided written informed consent.

## Results

### Demographic, biological and clinical characteristics of patients

The main socio-demographic and clinical characteristics of the enrolled patients are reported in Table 1. There were no differences between the groups at the study inclusion.

**Table 1:** Patient characteristics and biological parameters on the day of pharmacokinetic monitoring

Patient characteristics	Group RBT 150 mg TPW (n = 9)	Group RBT 300 mg TPW (n = 7)	p
Age (years)	36.3 ± 6.70	34.7 ± 6.92	0.643
Sex			
Male	6/9	2/7	0.131
Female	3/9	5/7	
Weight (kg)	51 ± 11.88	49.7 ± 8.80	0.832
Body mass index (BMI)	18.0 ± 3.74	17.1 ± 2,37	0.604
Dose of lopinavir (mg)/kg/12 h	8.07 ± 1.41	8. 51 ± 1.44	
Dose of ritonavir (mg)/kg/12 h	2.02 ± 0.35	2.13 ± 0.36	
Laboratory parameters			
Haemoglobin (g/dL)	10.3 ± 3.58	9.8 ± 1.45	0.802
Leucocytes (10^3^/mL)	4960 ± 3050	3900 ± 1643	0.554
Neutrophils (10^3^/mL)	3890 ± 1935	3956 ± 2985	
Lymphocytes (10^3^/mL)	1420 ± 837	1375 ± 801	0.937
Monocytes (10^3^/mL)	260 ± 89	225 ± 189	0.722
AST (U/L)	61.8 ± 20.31	48.2 ± 37.44	0.507
ALT (U/L)	49.4 ± 34.28	30 ± 22.31	0.363
Creatinine (µmol/L)	102.2 ± 27.54	106.1 ± 62.01	0.926
Total cholesterol (mmol/L)	162 ± 29.10	104.3 ± 58.44	0.200
HDL cholesterol (mmol/L)	24.3 ± 10.26	23 ± 9.89	0.894
Amylasaemia (U/L)	116.6 ± 18.03	98 ± 56.78	0.616
Total bilirubin (µmol/L)	6.2 ± 2.70	9.2 ± 4.80	0.360
Direct bilirubin (µmol/L)	0.87 ± 1.02	2.75 ± 2.33	0.213
Lymphocytes TCD4 (cells/µL)	221.1 ± 154.75	285.8 ± 175.39	0.446
Type of tuberculosis			
SPPT	7/9	7/7	0.475
SNPT	2/9	0/7)	–
WHO HIV stage			
Stage 2	1/9	0/7	0.562
Stage 3	8/9	7/7	

*TPW* three times per week, *h* hour, *AST* aspartate aminotransferase, *AST* alanine transaminase, *HDL* high-density lipoprotein, *SNPT* smear-negative pulmonary tuberculosis, *SPPT* smear-positive pulmonary tuberculosis

### Plasma concentration and pharmacokinetic parameters of lopinavir

**Fig. 1:**
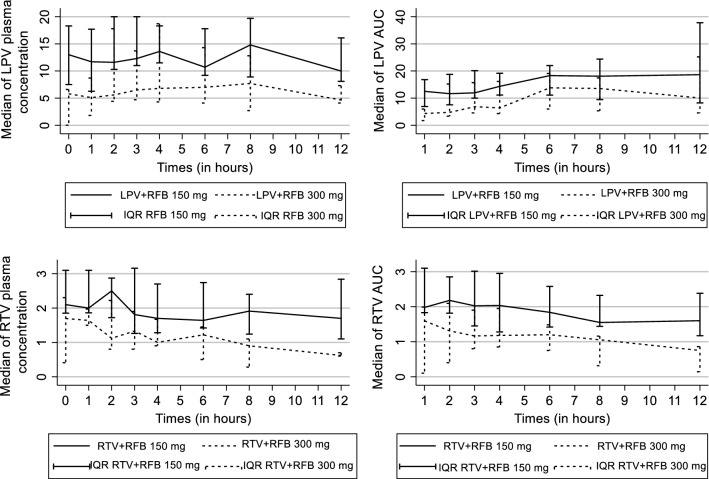
Lopinavir (LPV) and ritonavir plasma concentrations and area under the plasma concentration–time curve (AUC) in HIV-1-infected patients who used combination lopinavir/ritonavir twice daily with rifabutin 150 mg three times per week or rifabutin 300 mg three times per week. Data are presented as the medians with the inter quartile range. *RBT* rifabutin, *TPW* three times per week, *LPV/RTV* lopinavir/ritonavir, *IQR* interquartile range, *AUC*_*0–12h*_ area under the plasma concentration–time curve within the time span t_0_ to t_12_

As shown in Table 2 and Fig. 1, an RBT dosage of 300 mg thrice weekly resulted in a reduction of LPV plasma concentrations, C_max_ and AUC compared to an RBT dosage of 150 mg thrice weekly but the difference was not statistically significant. Furthermore, the average LPV concentrations at the end of the dosage intervals (C_0_) were 13 μg/mL for patients in arm A and 5.8 μg/mL for those in arm B (p = 0.044).

**Table 2:** Lopinavir (LPV) and ritonavir pharmacokinetic parameters in HIV-1-infected patients who used combination lopinavir/ritonavir twice daily with rifabutin 150 mg three times per week or rifabutin 300 mg three times per week

Pharmacokinetic parameters	Lopinavir 400 mg pharmacokinetic profile when used with RBT 150 mg or 300 mg			Ritonavir 100 mg pharmacokinetic profile when used with RBT 150 mg or 300 mg		
	LPV/RTV (400 mg/100 mg) twice daily with RBT 150 mg TPW (n = 9) (median and IQR)	LPV/RTV (400 mg/100 mg) twice daily with RBT 300 mg TPW (n = 7) (median and IQR)	p value	LPV/RTV (400 mg/100 mg) twice daily with RBT 150 mg TPW (n = 9) (median and IQR)	LPV/RTV (400 mg/100 mg) twice daily with RBT 300 mg TPW (n = 7) (median and IQR)	P value
C_0_ (µg/mL)	13 (7.5–18.3)	5.8 (0.01–6.6)	0.044	2.1 (1.8–3.1)	1.6 (0.4–2.3)	0.914
C_12_ (µg/mL)	10 (8.1–16.1)	4.6 (4.1–7.3)	0.170	1.7 (1.1–2.8)	0.6 (0.6–0.7)	0.265
C_max_ (µg/mL)	20 (11.5–21.4)	7.7 (5–19.5)	1.000	2.7 (1.9–3.1)	1.5 (0.9–2.4)	0.313
T_max_ (h)	4 (3–4)	3 (2–6)	1.000	3 (2–4)	2 (2–4)	0.313
CL (L/kg/h)	0.08 (0.05–0.10)	0.14 (0.09–0.18)	0.313	0.16 (0.11–0.18	0.29 (0.15–0.54)	0.313
AUC_0–12h_	111.8 (67.4–150.4)	69.9 (38.4–104.8)	0.313	12.7 (10.8–18.5)	6.6 (4.6–12.2)	0.313

Data are presented as the medians with the range in parentheses

*RBT* rifabutin, *TPW* three times per week, *LPV/RTV* lopinavir/ritonavir, *C*_*Tn*_ drug plasma concentration at the specified time, *IQR* interquartile range, *C*_*max*_ maximum (peak) plasma drug concentration, *T*_*max*_ time to reach maximum (peak) plasma concentration following drug administration, *C*_*0*_ plasma drug concentration before the morning dose; *C*_*12*_ plasma drug concentration before the evening dose (12 h post-dose), *AUC*_*0–12h*_ area under the plasma concentration–time curve within the time span t_0_ to t_12_

The AUC analysis of LPV showed a reduction between 150 mg RBT and 300 mg RBT. The AUC_0–12_ of LPV was 111.8 (IQR: 67.4–150.4) μg h/mL in patients treated with RBT 150 mg versus 69.9 (IQR: 38.4–104.8) μg/mL in those treated with RBT 300 mg thrice weekly (p = 0.313). However, the clearance of LPV appeared to be more important among patients receiving higher RBT doses.

Data from individual plasma concentrations of LPV in patients in the RBT 300 mg group suggest that the LPV C_0_ were lower than 4 μg/mL in three patients (0.01 μg/mL in two patients and 1.62 μg/mL in one patient) and the concentration after 12 h was least than 1 μg/mL in two patients treated with RBT 300 mg (Table 3). In the group of patients treated with RBT 150 mg thrice weekly, with the exception of a patient who had a plasma concentration of 1 μg/mL at the 12th h, all patients had sufficiently high plasma concentrations (> 4 μg/mL) including C_0_ to C_12_ (Tables 3, 4).

**Table 3:** Individual LPV plasma concentrations in patients treated with RBT 150 mg TPW or RBT 300 mg TPW

Patients	Sex	Age (years)	Weight (kg)	C_0_ (µg/mL)	C_1_	C_2_	C_3_	C_4_	C_6_	C_8_	C_12_	AUC_0–12_ (µg h/mL)	Cl (L/h/kg)	C_max_	T_max_
First group (RBT 150 mg TPW)															
Patient1	F	39	50.5	11.1	2.7	11.5	4.9	9.8	4.7	19.7	1	109.8	0.07	9.8	4
Patient3	M	36	56.8	18.3	15.3	21.4	20	13.6	9.2	8.9	10	151.3	0.05	21.4	2
Patient5	M	43	45.2	13	19.4	20	17.2	21.7	14	15.2	16.9	203.1	0.04	20	2
Patient7	M	32	44	13.3	11.7	11.6	12.3	16.4	23.7	21.6	15.7	111.8	0.08	23.7	6
Patient9	F	39	36	18.3	17.7	19.8	20.4	18.8	17.8	14.8	16.1	126.5	0.09	20.4	3
Patient11	F	33	57.6	7.5	8	10.3	10.8	11.5	10.7	8.2	8.3	67.4	0.11	11.5	4
Patient13	M	34	55	7.35	6.26	8.8	11.2	12.2	8.74	6.23	8.1	61.2	0.12	12.2	4
Patient15	M	24	52	22.1	23.2	20.4	20	18.3	25.7	23.7	16.2	150.4	0.05	25.7	6
Patient17	M	47	60	6.26	5.7	5.8	11	8.3	9.4	9	5.84	55.3	0.12	11	3
Second group (RBT 300 mg TPW)															
Patient2	F	33	40	0.01	1.1	5.6	13.7	15.1	10.2	9.1	4.4	99.5	0.10	15.1	4
Patient4	M	44	55	5.8	2.3	5	3.7	4.3	4.1	2.7	0.9	38.4	0.19	5	2
Patient6	F	33	40.1	1.62	1.82	4.36	5.85	6.8	7	7.7	4.9	69.9	0.14	7.7	8
Patient8	M	32	60.8	6	6	7.2	6.5	6.3	5.7	5	4.1	41.7	0.16	7.2	2
Patient10	F	32	40.2	0.01	8.7	22.3	23.9	19.2	14.3	12.8	7.3	104.9	0.09	23.9	3
Patient12	F	25	49.2	12.1	12.6	17.8	13.6	18.7	19.5	15.4	12.2	109.7	0.07	19.5	6
Patient14	F	44	52	6.6	5.1	4.42	4.7	3.94	2.7	1	Low	25.2	0.30	4.7	3

### Plasma concentration and pharmacokinetic parameters of ritonavir

The RTV plasma concentrations were reduced by nearly half in patients receiving RBT 300 mg compared to those on RBT 150 mg (Table 2; Fig. 1). The AUC_0–12_ of the RTV in arm A was 12.7 (IQR: 10.8–18.5) μg h/mL versus 6.6 (IQR: 4.6–12.2) μg h/mL observed in arm B but the difference was not statistically significant. There was no significant change in the T_max_ and the clearance of RTV between the two study groups. Regarding individual plasma concentrations of RTV, one patient treated with RBT 300 mg had a C_0_ below the limit of quantification and another one had a C_12_ below this limit (Table 4).

**Table 4:** Individual RTV plasma concentrations in patients treated with RBT 150 mg TPW or RBT 300 mg TPW

Patients	Sex	Age (years)	Weight (kg)	C_0_ (µg/mL)	C_1_	C_2_	C_3_	C_4_	C_6_	C_8_	C_12_	AUC_0–12_ (µg h/mL)	Cl(L/h/kg)	C_max_	T_max_
First group (RBT 150 mg TPW)															
Patient1	F	39	50.5	1.2	0.4	1.7	0.64	1.28	0.7	2.4	0.2	7.8	0.25	1.28	4
Patient3	M	36	56.8	2.1	1.86	2.5	0.4	0.5	1	1	1.33	8.9	0.19	1.86	1
Patient5	M	43	45.2	1.43	2	2.24	1.81	2.26	1.42	1.5	1.7	12.8	0.17	2.26	4
Patient7	M	32	44	2.1	1.56	1.56	1.26	1.3	2.74	1.91	1.7	12.2	0.18	2.74	6
Patient9	F	39	36	2.96	2.5	2.87	3.16	2.74	2.42	1.93	2.84	18.5	0.15	3.16	3
Patient11	F	33	57.6	7.5	8	10.3	10.8	11.5	10.7	8.2	8.3	67.4	0.03	11.5	4
Patient13	M	34	55	1.85	1.91	1.72	1.65	1.2	1.64	1.24	1.1	10.8	0.17	1.91	1
Patient15	M	24	52	4.11	3.8	3.6	3.5	2.7	3.3	2.8	3.6	23.5	0.08	3.6	2
Patient17	M	47	60	3.1	3.1	2.6	2.9	1.7	1.5	1.2	0.6	14.8	0.11	2.9	3
Second group (RBT 300 mg TPW)															
Patient2	F	33	40	Low	Low	1	1.33	1.44	1.22	1.1	0.62	6.4	0.39	1.44	4
Patient4	M	44	55	0.41	0.37	0.4	0.3	0.38	0.3	0.28	low	2.2	0.81	0.4	2
Patient6	F	33	40.1	0.2	Low	0.8	0.8	0.9	0.8	0.9	0.6	4.6	0.54	0.9	4
Patient8	M	32	60.8	2.72	1.97	2.22	1.41	0.95	1.45	0.66	0.53	10.3	0.16	2.22	2
Patient10	F	32	40.2	1.84	2.14	2.43	2.4	1.67	1.3	1	0.7	12.2	0.20	2.43	2
Patient12	F	25	49.2	1.55	1.64	1.9	1.9	2	2.5	2	1.86	13.6	0.15	2.5	6
Patient14	F	44	52	2.3	1.5	1.12	1.2	1	0.5	0.13	Low	6.6	0.29	1.5	1

*RBT* rifabutin, *TPW* three times per week, *LPV* lopinavir, *RTV* ritonavir, *Cn* drug plasma concentration at the specified time, *C*_*max*_ maximum (peak) plasma drug concentration, *T*_*max*_ time to reach maximum (peak) plasma concentration following drug administration, *C0* plasma drug concentration before the morning dose, *C12* plasma drug concentration before the evening dose (12 h post-dose)

## Discussion

Our study evaluated the pharmacokinetics of lopinavir and ritonavir in TB and HIV co-infected patients treated with RBT 150 mg or RBT 300 mg thrice weekly. The results show that treatment with RBT 300 mg decreases the exposure parameters of LPV and RTV (C_max_, C_0_, AUC_0–12_) more than treatment with RBT 150 mg. Although the median plasma concentrations of LPV remained above the therapeutic threshold, the concentrations were inadequate for some patients in our study. Importantly, the C_0_ medians of LPV were higher among patients receiving RBT 150 mg, at 13 μg/mL versus 5.8 μg/mL. The minimum plasma concentration of LPV that is recommended to reach therapeutic efficacy in ART-naïve adult patients is at least 1 μg/mL [24]. However, the impact of the minimum concentration (C_min_) of LPV on mutations and treatment failures was evaluated in the KALEPHAR study, which set the minimum intracellular and plasma concentrations at 8 and 4 μg/mL, respectively [29]. When considering the individual results in our study, four patients in the RBT 300 mg thrice weekly group and one patient in the RBT 150 mg thrice weekly group had C_0_ or C_12_ below this target (0.01 to 1.62 μg/mL). Matteelli et al. [25] found that the plasma concentration of LPV in TB/HIV co-infected patients was not affected by low RBT dosages (150 mg TPW). Our results suggest that the standard dosage of twice daily LPV/RTV 400/100 mg may be low for TB-HIV dually infected patients receiving RBT 300 mg TPW. The C_0_ cut-off of LPV associated with virologic failure of HIV treatment has yet to be accurately defined in ART-naive subjects such as those enrolled in our study, but according to Boffito et al. [6], the LPV C_0_ for optimal efficacy in HIV-infected patients on ART should be greater than 5.7 μg/mL. The interaction between LPV and antituberculosis drugs of the rifamycin class has been widely described [3, 10, 18, 30], but the interaction is likely less pronounced with RBT compared to RIF [14, 17]. Indeed, rifamycins are potent inducers of the CYP450 enzymatic system, and protease inhibitors (PIs) are metabolized by the CYP450 enzyme system, particularly by CYP3A4. Co-administration of rifamycin and PI leads to a reduction in the plasma concentration of IP [5]. These interactions may lead to an increased risk of TB drug toxicity [31, 32], failure of HIV treatment, and potential development of drug resistance [29].

In our study, as observed for LPV, the RTV-related pharmacokinetic parameters (C_max_ and AUC_0–12_ as well as C_0_) were lower for patients on RBT 300 mg TPW than those on RBT 150 mg TPW. Ritonavir is a PI used to increase and maintain plasma concentrations of LPV for a long time or at least until the next dose [33, 34]. It facilitates the absorption of other PIs, including through inhibition of enteric enzymes that play a role in degrading this drug class, and liver enzymes involved in PI metabolism. The reduction of plasma concentration of ritonavir on RBT 300 mg arm compare to RBT 150 mg arm observed in our study is probably due to the pronounced interaction with higher dose of RBT and, as expected, resulting in a greater reduction in the plasma concentration of RTV and a decrease in its potentiating effect on LPV [35, 36].

Our study has some limitations. First, the number of enrolled patients was very small, which may limit the generalizability of our findings. Second, a proper assessment of the impact of RBT-induced reduction in plasma concentration of LPV/RTV was not possible due to the absence of a control group. Third, our study did not evaluate the effectiveness of the two treatment regimens on the virological response. Despite these limitations, our findings provide interesting pharmacological insights that could encourage future studies to assess the virological efficacy and the incidence of adverse events associated with each of the therapeutic combinations on a larger number of patients.

## Conclusion

The pharmacokinetic of LPV and RTV was found to be highly variable when coadministrated with RBT 150 mg or 300 mg three times per week. Although therapeutic drug monitoring to ensure adequate LPV plasma concentrations when co-administered with RBT can be suggested in the high resource settings, it is not applicable in developing countries where HIV and tuberculosis are endemic. There is a need for specific large study to verify clinical and virological effects of the reduction of LPV, especially when it is coadministrated with RBT 300 mg TPW to prevent viral resistance in response to under-dosing of LPV.

## Data Availability

The datasets used and/or analysed during the current study are available from the corresponding author on reasonable request.

## Abbreviations

AIDS: acquired immune deficiency syndrome
ART: antiretroviral therapy
ARV: antiretroviral
AUC: area under the curve
CDC: Centers for Disease Control and Prevention
CERS: Ethics Committee for Health Sciences
C_max_: maximum concentration
DOTS: directly observed treatment short-course
d-RBT: 25-*O*-desacetyl-rifabutin
EOD: every other day
HIV: human immunodeficiency virus
IQR: interquartile range
LPV: lopinavir
RTV: ritonavir
PI: proteases inhibitor
RBT: rifabutin
T_max_: time at which the C_max_ is observed
